# Phylogeography of a migratory songbird across its Canadian breeding range: Implications for conservation units

**DOI:** 10.1002/ece3.3170

**Published:** 2017-06-28

**Authors:** Samuel Haché, Erin M. Bayne, Marc‐André Villard, Heather Proctor, Corey S. Davis, Diana Stralberg, Jasmine K. Janes, Michael T. Hallworth, Kenneth R. Foster, Easwaramurthyvasi Chidambara‐vasi, Alexandra A. Grossi, Jamieson C. Gorrell, Richard Krikun

**Affiliations:** ^1^ Department of Biological Sciences University of Alberta Edmonton AB Canada; ^2^ Environment and Climate Change Canada Yellowknife NT Canada; ^3^ Département de biologie, chimie et géographie Université du Québec à Rimouski Rimouski QC Canada; ^4^ Department of Renewable Resources University of Alberta Edmonton AB Canada; ^5^ Migratory Bird Center Smithsonian Conservation Biology Institute National Zoological Park Washington DC USA; ^6^ Owl Moon Environmental Inc. Fort McMurray AB Canada; ^7^ Biology Department Vancouver Island University Nanaimo BC Canada; ^8^ Lesser Slave Lake Bird Observatory Slave Lake AB Canada

**Keywords:** genetics, light‐level geolocator, microsatellites, migration, Ovenbird, paleohindcast models, population structure, *Seiurus aurocapilla*, syringophilid quill mites

## Abstract

The objectives of this study were to describe and evaluate potential drivers of genetic structure in Canadian breeding populations of the Ovenbird, *Seiurus aurocapilla*. We performed genetic analyses on feather samples of individuals from six study sites using nuclear microsatellites. We also assessed species identity and population genetic structure of quill mites (Acariformes, Syringophilidae). For male Ovenbirds breeding in three study sites, we collected light‐level geolocator data to document migratory paths and identify the wintering grounds. We also generated paleohindcast projections from bioclimatic models of Ovenbird distribution to identify potential refugia during the last glacial maximum (LGM, 21,000 years before present) as a factor explaining population genetic structure. Birds breeding in the Cypress Hills (Alberta/Saskatchewan) may be considered a distinct genetic unit, but there was no evidence for genetic differentiation among any other populations. We found relatively strong migratory connectivity in both western and eastern populations, but some evidence of mixing among populations on the wintering grounds. There was also little genetic variation among syringophilid mites from the different Ovenbird populations. These results are consistent with paleohindcast distribution predictions derived from two different global climate models indicating a continuous single LGM refugium, with the possibility of two refugia. Our results suggest that Ovenbird populations breeding in boreal and hemiboreal regions are panmictic, whereas the population breeding in Cypress Hills should be considered a distinct management unit.

## 1 INTRODUCTION

Over the past several decades, many species of songbirds breeding in North America have undergone substantial population declines (NABCI, 2016). In the case of declining species with extensive distributions and geographical variation in habitat selection and trends, many authors have argued for spatially explicit conservation units to ensure effective population‐level conservation plans (Carter, Hunter, Pashley, & Rosenberg, 2000; Rosenberg & Blancher, 2005; Rushing, Ryder, Scarpignato, Saracco, & Marra, 2016). However, many different population concepts have been used to delineate conservation units for migratory songbirds (e.g., global, breeding, regional populations, and subpopulations) and clearer guidance based on genetic and demographic data is required to inform conservation efforts (e.g., Moritz, 1994; Rushing et al., 2016).

In theory, one of the most direct approaches to identify conservation units is to find individuals sharing unique phenotypic and genotypic attributes in a defined area. If these unique attributes exist, that would suggest that the population is locally adapted and regionally‐ shared phenotypic attributes and subtle morphological characteristics have led to the recognition of subspecies (Patten & Unit, 2002; Phillimore & Owens, 2006). It has been argued that genetic assessments of subspecies should focus on putatively adaptive markers rather than neutral markers to distinguish among phenotypes (Patten, 2015; but see Moritz, 1999; Manthey, Klicka, & Spellman, 2011). However, genetic structure analyses across species ranges have been conducted for only a few migratory songbird species (e.g., Colbeck, Gibbs, Marra, Hobson, & Webster, 2008; Ralston & Kirchman, 2012) and use of molecular markers coding for specific phenotypic traits is often not an option for most nonmodel species (but see Van Bers et al., 2012; Ruegg et al., 2014).

Genetic structure, isolation, and drift can all result from historical processes. For example, Pleistocene glaciations are thought to have led to vicariance and subsequent speciation among northern avifauna (Johnson & Cicero, 2004; Lovette, 2005; Weir & Schluter, 2004). Although a single contiguous boreal forest glacial refugium in the southeastern United States is generally recognized (Dyke, 2005; Jackson & Overpeck, 2000; Overpeck, Webb, & Webb, 1992), wide‐ranging boreal‐breeding bird species could have also had multiple geographically isolated refugia across North America during the Pleistocene epoch, leading to genetic and behavioral divergence. Molecular evidence from a few wide‐ranging migratory species like Swainson's Thrush (*Catharus ustulatus*), Wilson's Warbler (*Cardellina pusilla*), and Yellow Warbler (*Setophaga petechia*) supports the existence of multiple isolated glacial refugia ~20,000 years ago (Clegg, Kelly, Kimura, & Smith, 2003; Milot, Gibbs, & Hobson, 2000; Ruegg, Hijmans, & Moritz, 2006).

Such historical isolation may explain current differences in migratory paths and wintering areas. However, the importance of historical versus current barriers and contemporary demographic processes on limiting migration and dispersal pathways—and, ultimately, influencing the persistence of distinct genetic structure—is relatively unexplored (but see Ruegg et al., 2006; Milá, McCormack, Castaneda, Wayne, & Smith, 2007; Delmore, Fox, & Irwin, 2012). Thus, the degree to which populations are demographically (social interactions via natal or breeding dispersal) or genetically isolated is effectively unknown for most migratory songbirds.

For migratory species, understanding the geographical connections between different phases of the annual cycle (i.e., migratory connectivity) is essential for effective conservation (Marra, Hunter, & Perrault, 2011). When migratory connectivity is strong, wintering and breeding populations are considered as discrete units. Weak migratory connectivity implies considerable mixing among populations on the wintering grounds or large‐scale dispersal among breeding areas (Webster, Marra, Haig, Bensch, & Holmes, 2002). Strong migratory connectivity makes it easier to assess the efficacy of full life‐cycle management actions (e.g., Taylor & Stutchbury, 2016). With the development of tracking technologies, migratory connectivity can now be quantified using light‐level geolocation, radiotelemetry, or GPS tags (Hallworth & Marra, 2015; Stutchbury et al., 2009; Woodworth, Mitchell, Norris, Francis, & Taylor, 2015).

Even minimal movement among bird populations can result in genetic panmixia (e.g., Cortes‐Rodriguez, Sturge, & Omland, 2016), further complicating the distinction of conservation units. However, cryptic genetic structure in such apparent panmixia can often be identified using parasites, mutualists, and commensals. Symbionts of birds have been shown to be useful biological markers for resolving host evolutionary history, population structure, and migratory patterns because of their shorter generation time compared to their hosts (Bruyndonckx, Biollaz, Dubey, Goudet, & Christe, 2010; Palopoli et al., 2015; Whiteman, Kimball, & Parker, 2007). Variation in symbiont assemblages or in genetic structure of particular species of symbionts can potentially provide finer resolution of host structure than the hosts themselves. However, not all symbionts are equally likely to reflect host population structure. Quill mites (Acariformes, Prostigmata, Syringophilidae) may be good indicators of host population structure, as they have no free‐living stage, undergo several generations per year on one host (Kethley, 1971), and are transmitted among birds only through close physical contact (e.g., between mating individuals, parent‐offspring, among nestlings). Unlike some lice and skin mites (Epidermoptidae), quill mites have never been reported as phoretic on vagile parasitic flies (e.g., Harbison & Clayton, 2011).

This study focuses on the Ovenbird (*Seiurus aurocapilla*; Linnaeus), a forest songbird with three defined subspecies (AOU, 1957) based on morphology: *S. a. aurocapilla*, which breeds in the boreal and hemiboreal portions of the species’ breeding range, *S. a. cinereus* in the Midwestern United States to the southwestern Canadian border, and *S. a. furvior*, found exclusively in Newfoundland. Using light‐level geolocators, Hallworth, Sillett, Van Wilgenburg, Hobson, and Marra (2015) showed strong migratory connectivity and clear segregation of wintering areas for eastern and western breeding populations of *S. a. aurocapilla,* suggesting an east‐west segregation by migratory flyway, as presumed by band recoveries (Atlantic and Mississippi flyways, respectively; Porneluzi, Van Horn, & Donovan, 2011; but see La Sorte et al., 2014). Breeding populations at the western end of the species range are thought to use the distinct Pacific flyway (Porneluzi et al., 2011).

**Figure 1:**
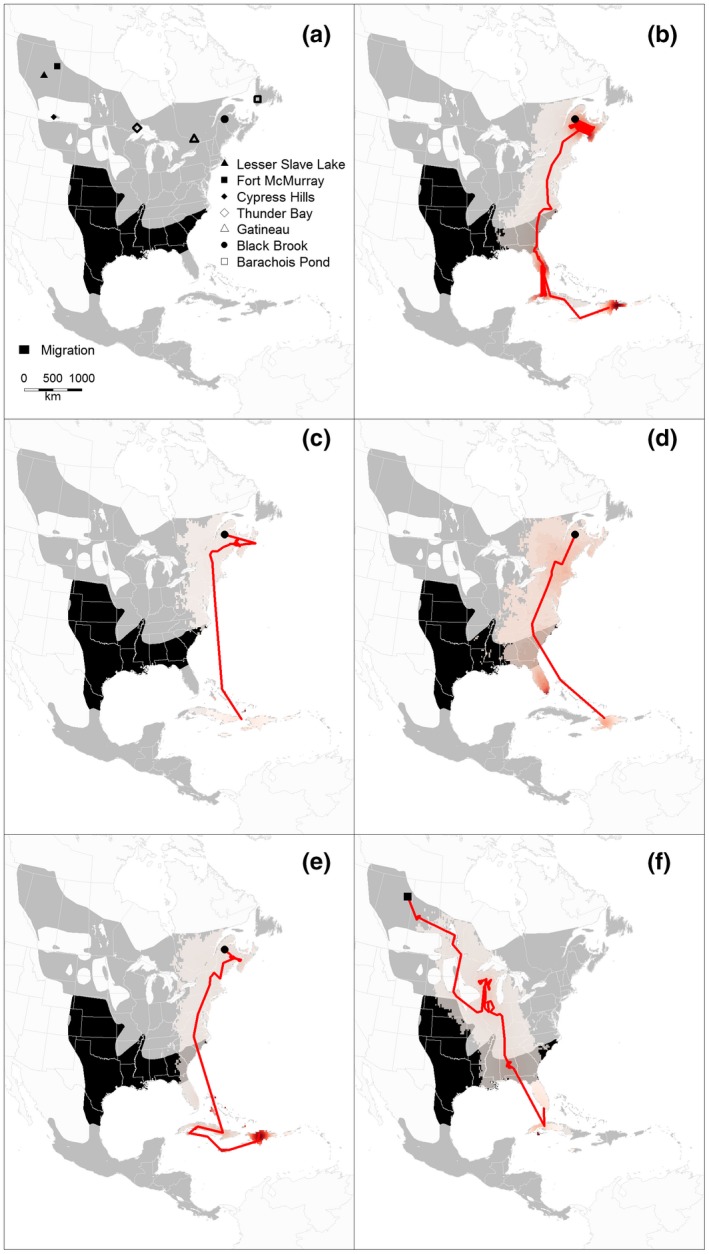
Study sites where feather samples from male Ovenbirds were collected and/or geolocators deployed (a). Also indicated are the breeding and wintering ranges according to BirdLife International and Nature Serve (2012). The red line depicts the median locations during spring migration and the light red error distribution represent 95% credible interval of the migration locations (b–f). The migratory routes and wintering grounds of *S. a. aurocapilla* individuals fitted with geolocators are indicated with a red color ramp. The map projection is North America Albers equal area

We used intrinsic and extrinsic markers to examine historical processes that may be responsible for the contemporary distribution of Ovenbird in Canada. Specifically, we conducted genetic analyses of birds and of their syringophilid quill mites sampled at six study sites. Results from genetic analyses were compared to those from light‐level geolocators deployed on individuals from populations in eastern and western Canada. In doing so, we evaluated the hypothesis that there would be population structure in Ovenbird across its Canadian breeding range. Population structure could occur among migratory flyways, whereby individuals from the western portion of the species’ Canadian breeding range would show strong migratory connectivity and be more genetically (host and symbionts) similar than those breeding in the eastern portion of the breeding range, despite the absence of physical barriers. Population structure could also occur both within and among migratory flyways as a result of finer‐scale migratory connectivity (e.g., leapfrog migration; Boulet & Norris, 2006). Alternatively, there could be enough dispersal movements (breeding and natal) among Ovenbirds in Canada to prevent genetic differentiation (host and symbionts; i.e., panmictic population). These alternatives were evaluated against paleoclimatic hindcasts of potential LGM refugia. Using these multiple lines of inquiry, we aimed to evaluate evidence for Ovenbird population structure that could in turn provide guidance for efficient population management and conservation.

## 2 MATERIALS AND METHODS

### 2.1 Study area and data collection

In 2012, territorial male Ovenbirds were captured in the Black Brook District, New Brunswick (47.50° N, 67.91° W), and in the Lesser Slave Lake Provincial Park at the Lesser Slave Lake Bird Observatory (LSLBO), Alberta (55.43° N, 114.82° W; Figure 1a). Individuals were captured using mist nets and playbacks of conspecific vocalizations and fitted with numbered aluminum bands. Feather samples (2 × 3rd retrix) were collected from 104 and 100 birds in Black Brook and LSLBO, respectively. In 2013, 31 additional feather samples were collected in Barachois Pond Provincial Park (Newfoundland; 48.48° N, 58.28° W), 29 in Gatineau, Quebec (45.63° N, 75.94° W), 26 in Thunder Bay, Ontario (48.47°N, 89.22° W), and 73 in Cypress Hills Interprovincial Park (49.66° N, 110.31° W), Alberta–Saskatchewan border (Figure 1a), for a total of 363 individual samples.

### 2.2 Bird genetic analyses

Microsatellite markers from other passerine species (Dawson, Gibbs, Hobson, & Yezerinac, 1997; Gibbs, Tabak, & Hobson, 1999; Stenzler, Fraser, & Lovette, 2004; Winker, Glenn, & Graves, 1999) were screened for polymorphism in Ovenbirds. In total, seven markers were selected for genotyping (Dawson et al., 1997; Stenzler et al., 2004) and an additional eight microsatellites were developed in this study. Full details of microsatellite development are in Appendix S1. All 15 microsatellites were scored using GeneMapper 4.0. micro‐checker 2.2 (Van Oosterhout, Hutchinson, Willis, & Shipley, 2004) was used to check for errors in the data and presence of null alleles. To ensure the neutrality of markers, we tested for linkage disequilibrium and deviation from Hardy–Weinberg equilibrium using genepop 4.2 (Rousset, 2008). Unbiased expected (*H*_E_) and observed heterozygosity (*H*_O_) were calculated using msa 4.05 (Dieringer & Schlötterer, 2003).

A principal coordinates analysis (PCoA) was performed in genalex 6.5 (Peakall & Smouse, 2012) using *G*′_ST_ estimates (Hedrick, 2005) to visualize population differentiation. To assess the level of population genetic structure present, we used structure 2.3 (Pritchard, Stephens, & Donnelly, 2000) with the following parameters: 10 iterations per *K* (i.e., clusters), a total of 500,000 MCMC and 50,000 burn‐in, admixture model, correlated allele frequencies, and testing *K* 1–8. STRUCTURE analyses were performed with and without location prior information (LOCPRIOR = 0 or 1; Hubisz, Falush, Stephens, & Pritchard, 2009) to improve detection of weak population structure. We used STRUCTURE HARVESTER (Earl & vonHoldt, 2012) to determine the optimal *K*, using both the natural log likelihood, Ln Pr (*K*), and second‐order rate of change of likelihood, Δ*K* (Evanno, Regnaut, & Goudet, 2005). Independent structure runs were aligned and plotted using CLUMPAK (Kopelman, Mayzel, Jakobsson, Rosenberg, & Mayros, 2015).

To complement assessments of population structure, we estimated the directional component of genetic divergence using DivMigrate (Sundqvist, Keenan, Zackrisson, Prodohl, & Kleinhans, 2016). To account for uneven sample sizes, we randomly subsampled from larger populations 10 times. The resulting 10 data sets comprised 22 individuals in each population, equal with the smallest sample size (Thunder Bay). DivMigrate was run with a 0.05 alpha, calculating *G*_ST_ statistics for network plots and displaying connections >0.5 in strength. *G*_ST_ matrix values were averaged across the 10 data sets.

### 2.3 Mite identification and genetic analyses

The calamus of each feather sample was examined for syringophilid mites using a dissecting microscope (see Fig. S2.1 in Appendix S2). In addition to the 363 feather samples used for bird genetic analyses, more samples from both LSLBO and Black Brook were examined. Calami with mites were cut and stored in 95% ethanol. DNA extractions were performed on calami with the mites in situ. If both calami from a single host contained mites, they were placed in the same tube and extracted as a single sample. See Appendix S2 for a detailed description of the amplification of a fragment of the cytochrome oxidase subunit I (COI) gene and technique used to slide mount 384 syringophilids from 20 host birds.

Contigs were assembled using seqman Ngen (DNASTAR Inc., Madison, WI, USA) and manually aligned in bioedit 7.2.0 (Hall, 1999). Genetic distances were calculated in mega 6 (Tamura, Stecher, Peterson, Filipski, & Kumar, 2013). Phylogenetic relationships were estimated under maximum‐likelihood (ML), maximum parsimony (MP), and neighbor‐joining (NJ) analysis, with 1,000 bootstrap iterations using paup* 4.0a146 (Swofford, 2002). The model of nucleotide substitution for analysis was selected using the Akaike Information Criterion of jModelTest (Darriba, Taboada, Doallo, & Posada, 2012; Guindon & Gascuel, 2003). The TIM2 + G model (Posada, 2003) was used for ML and NJ analysis. All sequences are available from GenBank (KY001935–KY001972). The syringophilid *Stibarokris phoeniconaias* Skoracki & O'Connor was chosen as an outgroup to root the Ovenbird syringophilid tree, and sequences for this species were obtained from GenBank (KF840699.1 and KF840700.1).

### 2.4 Light‐level geolocator analyses

In 2012, 13 individuals were fitted with archival light‐level geolocators in Black Brook and LSLBO (hereafter “geolocator,” 0.65 g, Mk20S, Lotek Wireless, Newmarket, Canada) (*n *=* *26). Each geolocator was glued to a leg‐loop harness that was fitted to a bird (after Rappole & Tipton, 1991). One or three color bands (one or two bands per leg) were also applied to each individual fitted with a geolocator for visual identification in the subsequent year based on unique color combinations.

In 2013, we retrieved four units from Black Brook and one from LSLBO, for a retrieval rate of 19%. We also deployed 20 geolocators at three additional locations: (1) LSLBO, (2) Fort McMurray, and (3) Cypress Hills Interprovincial Park (a total of 60 geolocators were deployed). In 2014, we retrieved seven geolocators (two from LSLBO, two from Fort McMurray, and three from Cypress Hills), for a retrieval rate of 12% (14% across the 2 years). In total, 19% (16/86) of individuals marked in year *x* returned in year *x *+* *1 (i.e., five birds could not be recaptured, loss their harness or geolocators failed to record data). Light data collected by the geolocators were converted to geographical locations (latitude and longitude) to document vernal migration and identify wintering grounds using the Solar/Satellite Geolocation for Animal Tracking package (“SGAT”; Wotherspoon, Sumner, & Lisovski, 2013; Sumner, Wotherspoon, & Hindell, 2009) in program R (see Appendix S3 in Supporting Information for more details). Raw light‐level data and sunrise/sunset times are available on Movebank.org (205765709).

### 2.5 Paleohindcast models

To identify likely glacial refugia for the Ovenbird, we developed a boosted regression tree distribution model (BRT; Elith, Leathwick, & Hastie, 2008) based on its current published range map (BirdLife International and NatureServe, 2012) and bioclimatic variables derived from 4‐km interpolated monthly climate data for the 1961–1990 normal period (Daly et al., 2008). We used this model to project refugia based on paleoclimate projections for the last glacial maximum (LGM).

As inputs to the distribution model, we sampled 10% of all 4‐km pixels across North America (*n* = 139,890) and intersected them with the range map to assign the presence/absence locations (prevalence = 0.256) as the dependent variable and with derived bioclimatic variables as independent predictors. We calculated seven bioclimatic variables chosen based on relevance to vegetation distributions (Hogg & Bernier, 2005), avoidance of extreme collinearity (Dormann et al., 2013), and a preference for seasonal over annual variables when they showed high correlations (Stralberg et al., 2015). These variables included extreme minimum temperature, chilling degree days, growing degree days, seasonal temperature difference, mean summer precipitation, climate moisture index, and summer climate moisture index (available as a data supplement to Stralberg et al., 2015).

We used the “dismo” (Hijmans, Phillips, Leathwick, & Elith, 2011) and “gbm” (Ridgeway, 2012) packages for R (R Core Team, 2014) to build a distribution model for the species, specifying a binomial distribution (“bernoulli” family). We used a stepwise procedure and 10‐fold crossvalidation to identify the optimal number of trees needed to maximize the mean deviance explained as recommended by Elith et al. (2008); we used a tree complexity of 3, learning rate of 0.01, and a bag fraction of 0.5.

To represent paleoclimate conditions, we obtained temperature and precipitation anomalies for 21,000 years BP (LGM) based on millennial equilibrium projections from two US global climate models (GCM): (1) the Community Climate Model (CCM1) developed by the National Center for Atmospheric Research (Kutzbach et al., 1998) and (2) the Geophysical Fluid Dynamics Laboratory (GFDL) model, from the National Oceanic and Atmospheric Administration. The monthly anomalies with respect to baseline climate variables (Roberts & Hamann, 2015; Stralberg et al., In press) were used as inputs to the BRT models to develop millennial‐scale hindcasts using the “raster” package for R (Hijmans & van Etten, 2012). Core habitats (both current range and LGM refugia) were defined as areas of predicted occupancy greater than 0.256 within the area covered by the training dataset, corresponding to the prevalence rate.

Finally, to compare climatic niches among subspecies, we repeated the above‐described process for mapped subspecies *S. a. aurocapilla*,* S. a. furvior,* and *S. a. cinereus* (adapted from American Ornithological Union, 1957). We also ran a principal component analysis on the three subspecies, using the “prcomp” function for R based on centered and scaled versions of the seven bioclimatic variables used to develop distribution models.

## 3 RESULTS

### 3.1 Bird genetics

Of the 363 feather samples collected, 339 were successfully genotyped at 12 or more loci. The presence of null alleles and linkage disequilibrium was not detected. Eight loci remained after the removal of seven loci deviating from HWE (see Table S3.2 in Appendix S3). The total number of alleles per locus ranged from 16 (Dpu16) to 31 (Dpu01). Population *H*_E_ was lowest for Cypress Hills and Thunder Bay (0.76) and highest for Gatineau and Barachois Pond (0.80; Table 1).

**Table 1:** Study site descriptions from six Ovenbird breeding populations including sample size (*N*), observed number of alleles per locus (*k*), unbiased observed (*H*_O_), and expected heterozygosity (*H*_E_) at eight microsatellite loci

Population^a^	*N*	*k ± SE*	*H*_O_ * ± SE*	*H*_E_ *± SE*
LSLBO, AB	91	17.0 ± 1.7	0.75 ± 0.06	0.79 ± 0.06
Cypress Hills, AB/SK	72	12.8 ± 1.6	0.72 ± 0.08	0.76 ± 0.07
Thunder Bay, ON	22	11.0 ± 1.4	0.72 ± 0.07	0.76 ± 0.08
Gatineau, QC	27	12.4 ± 1.4	0.80 ± 0.07	0.80 ± 0.05
Black Brook, NB	97	17.3 ± 1.6	0.73 ± 0.07	0.79 ± 0.06
Barachois Pond, NL	30	12.0 ± 1.4	0.76 ± 0.07	0.80 ± 0.06

a Birds from LSLBO, Thunder Bay, Gatineau, and Black Brook are from the *S. a. aurocapilla* subspecies, whereas those from Cypress Hills and Barachois Pond are from the *S. a. cinereus* and *S. a. furvior* subspecies, respectively.

**Figure 2:**
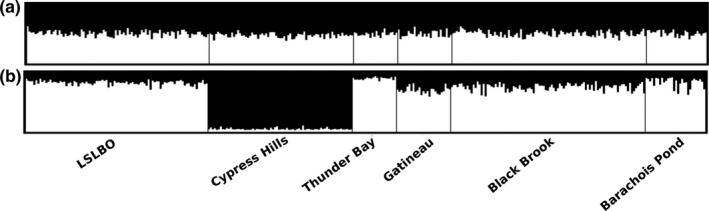
structure assignment of individuals to each genetic cluster for *K *=* *2 using eight microsatellites. Population structure was weak when sampling locations were not included as a prior in the analysis (a) and only detected when sampling locations were included (b)

**Figure 3:**
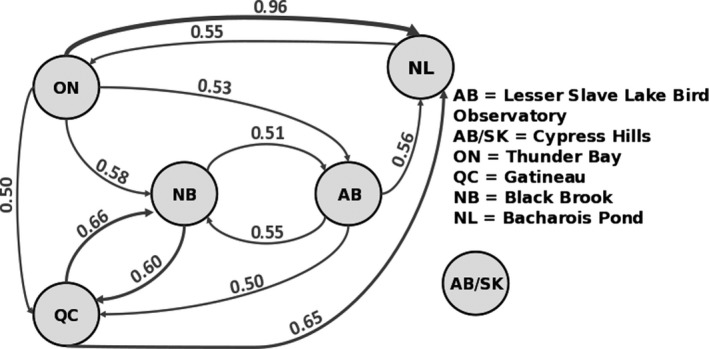
*G*_ST_ network showing the strength and direction of gene flow among populations

Estimates of genetic differentiation (*G*′_ST_) were low, ranging from −0.001 between Barachois Pond and Thunder Bay to 0.016 between Cypress Hills and Thunder Bay. The average pairwise *G*′_ST_ estimate was 0.007 (*SE* ± 0.002). Using location priors, STRUCTURE identified two distinct clusters (*K *=* *2) (Figure 2, see also Figs. S3.1 and S3.2 in Appendix S3). Under this model, Cypress Hills was distinguished from the remaining populations suggesting two genetically distinct populations. PCoA showed a similar pattern of genetic clustering (see Fig. S1.3 in Appendix S1). When Cypress Hills was removed from the STRUCTURE analysis, we found no evidence for further substructure despite retaining sampling location as a prior. DivMigrate suggested a similar pattern in which gene flow was present among all populations except Cypress Hills (Figure 3). Individual network plots for the 10 subsampled data sets are in Appendix S1.

### 3.2 Quill mite identification and genetics

Quill mites were present in at least one feather from 29 of 496 birds in 2012 and 19 of 379 in 2013. The morphology of all adult mites was consistent with *Betasyringophiloidus seiuri* (Clark) as illustrated in Bochkov & Galloway (2001) (see Skoracki, Spicer, & OConnor, 2016 for comments on taxonomy of this species). However, only 38 samples produced sequence data of high enough quality for phylogenetic analyses. The final alignment of COI was 613 bp in length with 68 variable sites of which 39 were parsimony informative. All analyses converged on the same topology (see Fig. S2.2 in Appendix S2). Due to the number of polytomies, there is little evidence of distinct clustering in host populations and very little variation among specimens. Since the mean genetic distance among the Cypress Hills specimens is larger than the mean distances between the Cypress Hills mites and those from other areas (Table S2.1 in Appendix S2), the Cypress Hills mites cannot be considered a distinct taxon (Čandek & Kuntner, 2015).

### 3.3 Light‐level geolocator analysis

**Figure 4:**
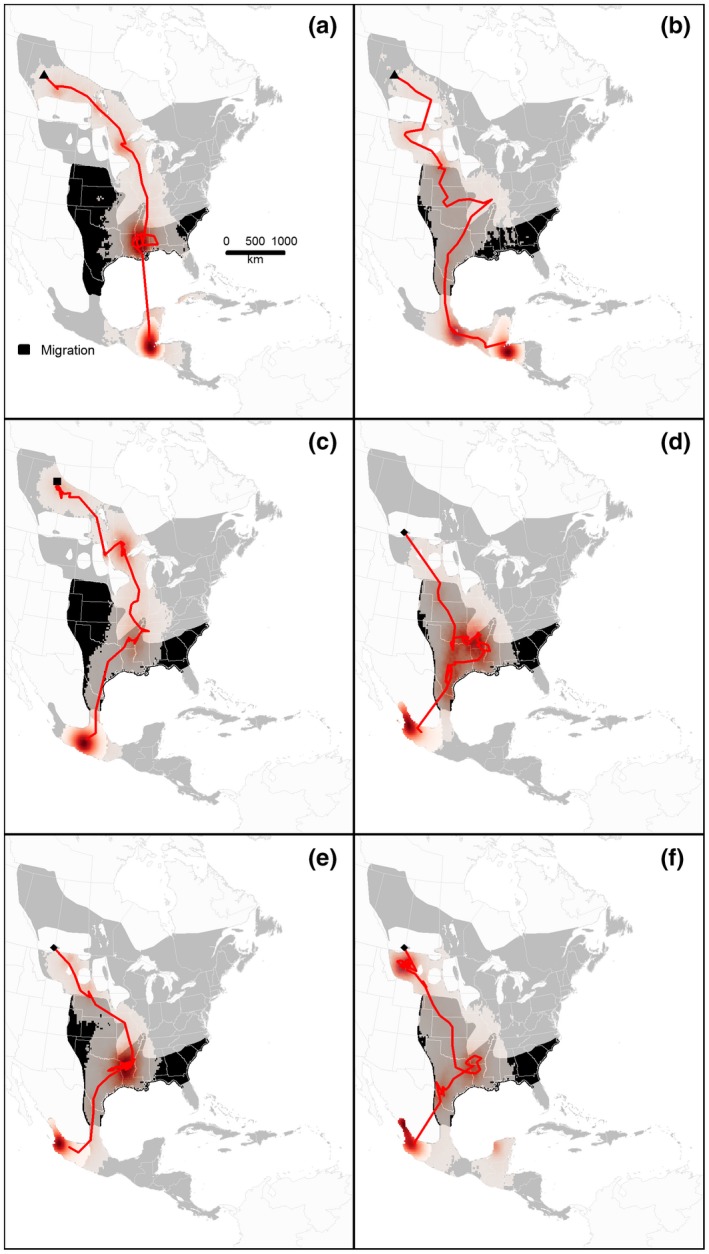
Wintering locations and spring migratory routes of *S.a. aurocapilla* (a–c) and *S. a. cinereus* individuals (d–f) fitted with geolocators. See Figure 1 legend for details. The map projection is North America Albers equal area

Geolocators recovered from New Brunswick (Black Brook) suggest that individuals from this population overwintered in Cuba, Hispaniola, or the Bahamas (Figure 1b–e). One individual captured in Alberta (Fort McMurray) overwintered in either Florida, USA, or Cuba (Figure 1f), while the other individuals (*n* = 6) from Alberta overwintered in Central America (Figure 4). Two individuals from the LSLBO population overwintered on the Yucatan peninsula (Mexico, Belize, or Guatemala), or in Honduras or El Salvador (Figure 4a,b), while another individual from the Fort McMurray population likely overwintered in the Mexican state of Guerrero (Figure 4c). The individuals from Cypress Hills likely overwintered in the Mexican states of Nayarit and/or Jalisco (Figure 4e,f).

### 3.4 Paleohindcast models

**Figure 5:**
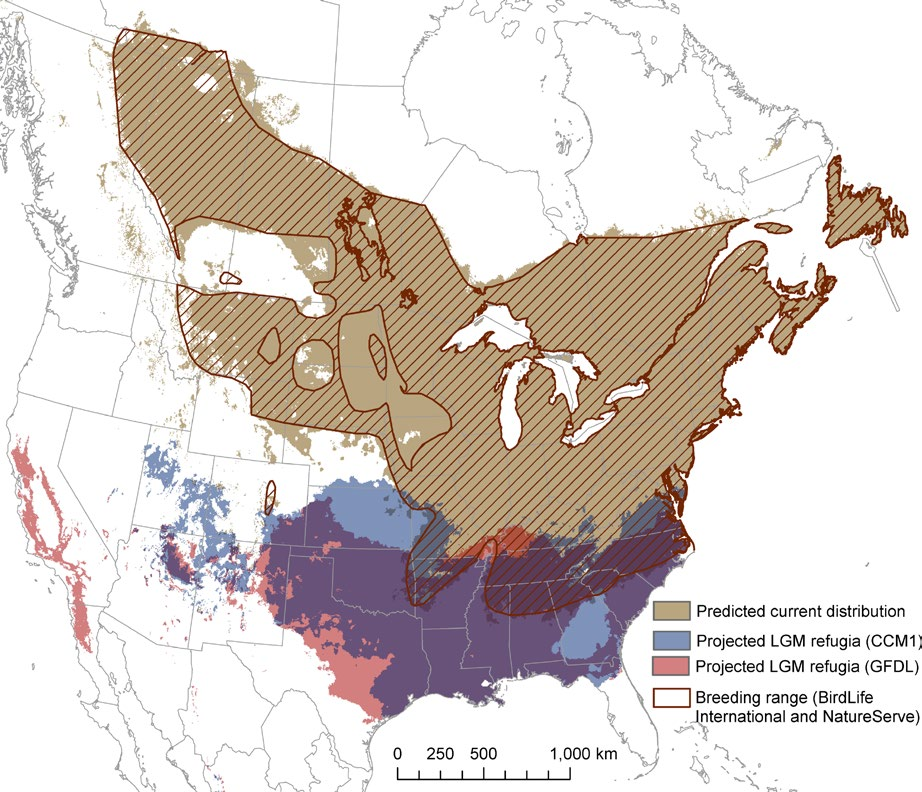
Projected climatic suitability of the Ovenbird’s breeding range during the last glacial maximum, based on two US global climate models (Community Climate Model [CCM1] and Geophysical Fluid Dynamics Laboratory [GFDL] model). Climatic suitability was determined using a threshold of 0.256 probability of occurrence. The map projection is North America Albers equal area

Predictive accuracy of the BRT distribution model was high: 94% as measured by the 10‐fold crossvalidation *R* ^2^. Spatial predictions from paleohindcasting based on both climate models suggest that the Ovenbird likely had a single contiguous refugium in the southeastern United States, overlapping with the southern limit of its current range, and possibly extending more patchily through the southwestern interior United States (Figure 5). According to the GFDL model, some suitable climate space was also available in California.

A principal components analysis revealed high overlap in climatic niche space for the three subspecies, with *S. a. furvior* completely overlapping the niche space of *S. a. aurocapilla* (Appendix S4). Hindcasts indicated that suitable LGM climate space for *S. a. cinereus* was identified in the southwestern interior United States and connected with the refugium for *S. a. aurocapilla*, while the narrow climate space occupied by *S. a. furvior* was likely not present at the LGM (Appendix S4).

## 4 DISCUSSION

Our results provide little evidence for genetic differentiation among Ovenbird populations in Canada, with the exception of the southwestern Cypress Hills population (*S. a. cinereus*). There was also limited genetic variation among symbionts (syringophilid mites) collected from all populations. These results are consistent with paleohindcast predictions from two different GCM, suggesting that a fairly contiguous glacial refugium was available in the southeastern United States 21,000 years BP, with the possibility of a separate western refugium (see also Stralberg et al., In press). We did, however, find support for differences in migratory patterns between eastern and western populations and those from northern versus southern Alberta, with evidence of relatively strong migratory connectivity within each of these three populations. The distinct genetic structure for *S. a. cinereus*, which could be maintained by the strong migratory connectivity exhibited by this population, provides some support for the current range delineation for this subspecies. However, no such evidence was found in support of *S. a. furvior*. Taken together, these results indicate that to be relevant, management units for migratory birds must be defined on the basis of detailed genetic data, but also information on migratory connectivity and glacial refugia.

### 4.1 Limited genetic isolation

Overall, Ovenbird populations displayed little genetic differentiation and weak population genetic structure, which is consistent with a single glacial refugium. However, the population in Cypress Hills in southern Alberta and Saskatchewan was consistently identified as genetically different. The low levels of genetic diversity within Cypress Hills suggest that this population may reflect historical founder effects after postglacial expansion. It may also have been separated from other populations at the LGM, according to our paleohindcasting results. Contemporary gene flow between Cypress Hills and the other populations is also limited. High gene flow, such as that identified among the five other populations, can lead to a homogenizing effect that can limit the ability to reliably detect underlying dispersal patterns among populations (Sundqvist et al., 2016). However, results from our additional analyses (PCoA and STRUCTURE) add support to the contention that the northern‐most population of *S. a. cinereus* (Cypress Hills) may warrant further study to determine whether its relative isolation could have a negative impact on the population.

In contrast, the five other populations display patterns suggestive of contemporary dispersal and panmictic gene flow that prevent the development of strong genetic structure. This observed panmictic boreal population is consistent with results from other studies on birds and other taxa, suggesting that relatively recent range expansions produce large areas of homozygosity and limited population structure (Cobben et al., 2011; Dyke, 2005; Hewitt, 2000). However, this level of genetic admixture likely contributes to fuzzy phenotypical and genetical boundaries among subspecies. Genetic information from a larger number of populations would be required for a more detailed assessment of the phylogeography of our focal species. It is also important to consider that the weak genetic structure we observed may reflect the resolution of markers used (e.g., Kimura et al., 2002; Ruegg et al., 2014).

### 4.2 Lack of population structure in symbionts

Syringophilids were found on at least one bird from each population, and all the specimens belonged to the same species (*B. seiuri*), resulting in no geographical variation in mite assemblages. There was also no genetic variation in syringophilids that was correlated with host localities. This may reflect host populations mixing on the wintering grounds or the more extensive breeding and natal dispersal movements generally observed in female songbirds compared to males (Greenwood, 1980; Greenwood & Harvey, 1982). It is also possible that there was genetic variation associated with host localities, but the marker used (COI) might not have reflected this. Other symbiont taxa might provide a higher resolution in host population structure. For example, members of the vane‐dwelling feather mite genera *Trouessartia* and *Proctophyllodes* were also collected from some rectrices (H. Proctor & A. Grossi, unpublished data), but at numbers too low to use as markers in this study. These mites may be more abundant on other feather types, and future studies could include their taxonomic and genetic analysis.

### 4.3 Migratory connectivity and population structure

With the exception of one individual breeding in Alberta that overwintered in either Florida or Cuba, migratory connectivity was strong, which is consistent with results from Hallworth et al. (2015). Few studies have documented weak migratory connectivity or population mixing in songbird species (e.g., Ruegg et al., 2014; Hobson & Kardynal, 2015; but see Finch, Butler, Franco, & Cresswell, 2017). Future studies should investigate the demographic implications of the relatively low mixing (9% of individuals; 1/11) reported in this study. Although geolocator data provide important information on species distribution on breeding and wintering grounds and their migratory routes, units have only been deployed on adult males (i.e., after hatch‐year) owing to higher site fidelity than females and hatch‐year birds. The implications of natal dispersal movements on the strength of migratory connectivity reported for songbirds (adults) remain unknown (Cresswell, 2014) and can only be quantified using other approaches such as stable isotopes, genetic analyses, or GPS tracking (e.g., Clegg et al., 2003; Kelly, Ruegg, & Smith, 2005).

Some studies have reported leapfrog migration in songbirds, where individuals breeding in the northern portion of the breeding range winter further south than southern‐breeding individuals (Bell, 1997; Boulet & Norris, 2006; Kelly, Atudorei, Sharp, & Finch, 2002). This migration system is consistent with the pattern observed in Ovenbirds breeding in Northern Alberta versus Cypress Hills to the south, with the latter consistently overwintering in northwestern portions of Mexico. Birds from Cypress Hills may also use the Pacific flyway, as individuals are being observed somewhat regularly in California during migration (Porneluzi et al., 2011). Different migration strategies among populations could lead to morphological differentiation (Winkler & Leisler, 1992; see also Desrochers, 2010). However, there was no significant difference in body weight and wing chord between birds breeding in Cypress Hills and those from northern Alberta (S. Haché, E. M. Bayne, and M.‐A. Villard, unpublished data). New Brunswick birds had significantly longer wings than those from the two western populations, but mean differences were unlikely to be biologically meaningful (0.8 and 1.4 mm, respectively; S. Haché, E. M. Bayne, and M.‐A. Villard, unpublished data). Other attributes such as wing shape, plumage color, and song characteristics should also be included in a more rigorous analysis of phenotypes across the breeding range (Patten, 2015).

### 4.4 Conservation implications

Population targets are being set via species‐level conservation plans such as Recovery Strategies and Action Plans for species at risk (Government of Canada, 2015). The Committee on the Status of Endangered Wildlife in Canada (COSEWIC) also regularly assesses subspecies (or varieties) and subpopulation status (COSEWIC, 2012). This hierarchical organization of individuals could be considered a simple concept applicable to a broader range of taxa, but such spatially‐explicit information is only available for a few migratory songbird species. Conservation efforts should focus on maintaining genetic diversity or evolutionarily significant units (e.g., Moritz, 1994, 1999; Palsboll, Bérubé, & Allendorf, 2006) and population dynamics through reproduction, survival, and functional/migratory connectivity (e.g., Rushing et al., 2016). Our results suggest that subspecies defined by arbitrary criteria do not provide a strong model for setting population management units. For example, conservation of Ovenbirds in Newfoundland (*S. a. furvior*) should be based on a larger area given the extensive gene flow that occurs with *S. a. aurocapilla*. In contrast, a subspecies‐based conservation strategy would be appropriate for *S. a. cinereus* in southern Alberta and Saskatchewan. Subspecies have been identified based on similar criteria for species of conservation concern (e.g., Rusty Blackbird, *Euphagus carolinus nigrans*, American Ornithological Union, 1957; and White‐winged Crossbill, *Loxia curvirostra percna*, Dickerman, 1987), and genetic analyses are required to inform population management plans. We encourage future studies such as this that employ multiple lines of evidence to identify different levels of population structure for conservation purposes.

## CONFLICT OF INTEREST

None declared.
