# Supplementary material for: Phylogeography of a migratory songbird across its Canadian breeding range: Implications for conservation units
Source: Ecol Evol. 2017 Jun 28;7(16):6078–88. doi: 10.1002/ece3.3170 (PMC5574796; doi:10.1002/ece3.3170)
Supplement: Supplementary file 1 [file ECE3-7-6078-s001.docx]

S. Haché, E.M. Bayne, M.-A. Villard, H. Proctor, C.S. Davis, D. Stralberg, J.K. Janes, M.T. Hallworth, K.R. Foster, E. Vasi, A.A. Grossi, J.C. Gorrell, and R. Krikun. Phylogeography of a migratory songbird across its Canadian breeding range: implications for conservation units. *Ecology and Evolution*.

**Appendix S1. Detailed description of the methods used to isolate, test, and genotype novel Ovenbird loci and additional supporting information for population structure analyses (tables and figures).**

Novel Ovenbird loci were isolated from a microsatellite enriched small insert library (Hamilton et al., 1999). Briefly, 15 ug of genomic DNA from a single individual was digested with Alu I and Nhe I, ligated to Super SNX linkers (Glenn & Schable, 2005) and hybridized to biotinylated (AC)_14_ and (AG)_14_ probes. Following stringency washes microsatellite enriched fragments were cloned into the XbaI site of pBluescript II (SK^+^) and transformed into E. coli XL1 blue competent cells. The inserts from 96 clones were amplified using T3 and T7 primers and sequenced in both directions using Big Dye 1.1 chemistry on an ABI 3730 DNA analyzer. Sequences were aligned using the SeqMan Pro application of the Lasergene suite (DNAstar) and consensus sequences were searched for microsatellite repeat motifs around which primers were designed using msatcommander 1.0.8-beta (Faircloth, 2008). Primers were designed and synthesized for 14 microsatellite bearing sequences and 19 loci from literature sources (Dpu 01, 03, 05, 15 and 16 from Dawson et al., 1997; Ccu 02, 04 and 28 from Gibbs et al., 1999; Lswu7, 9, 14 and 18 from Winker et al., 1999; and VeCr02, 05, 06, 07, 08, 10 and 14 from Stenzler et al., 2004).

These 33 loci were initially tested using eight individuals from across the species range. These amplification tests were carried out in 15µL reactions consisting of 1X PCR buffer (10mM Tris pH8.8, 0.1% Triton X-100, 50mM KCl, 0.16mg/mL BSA), 2.5mM MgCl_2_, 120µM each dNTP, 0.17 µM fluorescently labelled forward primer, 0.17µM reverse primer, 1U *Taq* DNA polymerase (Qiagen) and 2.5µL (approximately 50 ng) of extracted gDNA. Cycling conditions were 95^o^C for 5 minutes, 35 cycles of 95^o^C for 30 seconds, 55^o^C for 90 seconds, and 72^o^C for 30 seconds, and a final extension at 60^o^C for 30 minutes. Amplification reactions were pooled and loaded on an ABI 3730 DNA analyzer (ThermoFisher Scientific). Fragment sizes were determined relative to GeneScan-500 TAMARA (ThermoFisher Scientific) and scored using Genemapper.

Fifteen loci displaying clean, easily scored, polymorphic bands were genotyped in all individuals using three multiplexed 10 uL PCR reactions (Mix 1, 2and 3; Table S3.1) consisting of 1X Type-it Microsatellite PCR Master Mix (Qiagen), 1X primer mix (0.2 uM each), 0.5X Q-Solution (Qiagen), and 2.5µL of extracted gDNA. The methods and conditions for cycling, loading and sizing were identical to those used for testing.

We used the clustering method developed by Hubisz et al. (2009) and implemented in structure 2.3 (Pritchard et al., 2000) to investigate the presence of distinct populations. We used admixture models with correlated allele frequencies and either including or excluding original sampling location as prior information in separate models (LOCPRIOR=0 or 1). Ten iterations were run for each model, with 500,000 MCMC repetitions after a burn-in of 50,000 repetitions, testing up to six genetic clusters. We used structure harvester (Earl & vonHoldt, 2012) to determine the optimal number of genetic clusters, K, using the log likelihood, L(K), convergence between runs, and change in the second order of likelihood, ΔK (Evanno et al., 2005). Independent structure runs were then aligned and plotted using clumpak (Kopelman et al., 2015).

**References**

Dawson, R.J.G., Gibbs, H.L., Hobson, K.A. & Yezerinac, S.M. (1997) Isolation of microsatellite DNA markers from a passerine bird, the yellow warbler *Dendroica petechia* (the yellow warbler), and their use in population studies. *Heredity*, **78**, 506–514.

Earl, D.A. & vonHoldt, B.M. (2012) STRUCTURE HARVESTER: a website and program for visualizing STRUCTURE output and implementing the Evanno method. *Conservation Genetics Resources*, **4**, 359–361.

Evanno, G., Regnaut, S. & Goudet, J. (2005) Detecting the number of clusters of individuals using the software structure: a simulation study. *Molecular Ecology*, **14**, 2611–2620.

Faircloth, B.C. (2008) MSATCOMMANDER: detection of microsatellite repeat arrays and automated, locus specific primer design. *Molecular Ecology Resources*, **8**, 92–94

Gibbs, H.L., Tabak, L.M. & Hobson, K. (1999) Characterization of microsatellite DNA loci for a neotropical migrant songbird, the Swainson’s thrush (Catharus ustulatus). *Molecular Ecology*, **8**, 1551–1561.

Glenn, T.C. & Schable, N.A. (2005) Isolating microsatellite DNA loci. *Methods in Enzymology*, **395**, 202–222.

Hamilton, M.B., Pincus, E.L., Di Fiore, A. & Fleischer, R.C. (1999) Universal Linker and Ligation Procedures for Construction of Genomic DNA Libraries Enriched for Microsatelites. *BioTechniques*, **27**, 500–507.

Hubisz, M.J., Falush, D., Stephens, M. & Pritchard, J.K. (2009) Inferring weak population structure with the assistance of sample group information. *Molecular Ecology Resources*, **9**, 1322–1332.

Kopelman, N.M., Mayzel, J., Jakobsson, M., Rosenberg, N.A., Mayros, I. (2015) Clumpak: a program for identifying clustering modes and packaging population structure inferences across K*. Molecular Ecology Resources,* **15***, 1179*–*1191.*

Pritchard, J.K., Stephens, M. & Donnelly, P. (2000) Inference of population structure using multilocus genotype data. *Genetics*, **155**, 945–959.

Stenzler, L.M., Fraser, R. & Lovette, I.J. (2004) Isolation and characterization of 12 microsatellite loci from Golden-winged Warblers (*Vermivora chrysoptera*) with broad cross-taxon utility in emberizine songbirds. *Molecular Ecology Notes*, **4**, 602–604.

Winker, K., Glenn, T.C. & Graves, G.R. (1999) Dinucleotide microsatellite loci in a migratory wood warbler (Parulidae: Limnothlypis swainsonii) and amplification among other songbirds. *Molecular Ecology*, **8**, 1551–1561.

**Table S1.1**. Primer Sequences, repeat motifs, observed size range of alleles, multiplex reaction, source, and GenBank accession numbers for microsatellite loci used to genotype the Ovenbird.

| **Locus** | **Primer Sequence** | **Repeat Motif** | | **Size (bp)** | **Multi plex** | **Source** | **Accession Number** |
| --- | --- | --- | --- | --- | --- | --- | --- |
| Sau10 | F: TET-CATTTGCTTTAGACCTGTCC | AC(10) | | 233-269 | 2 | This study | KX901662 |
|  | R: GTTAACAATGTTTAGGCCTGTTG |  | |  |  |  |  |
| Sau15 | F: HEX-TAGGAAAACCAGGTACCATG | AC(11) | | 327-357 | 1 | This study | KX901663 |
|  | R: GTTCTTTTTGATGTTGCTTTGC |  | |  |  |  |  |
| Sau26 | F: TET-AAACAAGGTTTCCATTCCAG | AC(16) | | 212-256 | 1 | This study | KX901664 |
|  | R: GTTATGCTGTGATGACATTCAAC |  | |  |  |  |  |
| Sau38 | F: FAM-CTTAGGTCCTATTGCCAGTG | AC(11) | | 135-149 | 1 | This study | KX901665 |
|  | R: GTTGACAAAGTTACCCTACCTG |  | |  |  |  |  |
| Sau45 | F: FAM-GTTGGCAAAAGGAGAAGTAG | AC(17) | | 202-237 | 2 | This study | KX901666 |
|  | R: GTTACAGAAGGTAAATGGGGTG |  | |  |  |  |  |
| Sau46 | F: HEX-AAAGCATTGCACATCACAG | AC(13) | | 199-219 | 2 | This study | KX901667 |
|  | R:GTTAGCAAAATACAAGAGCAAGG |  | |  |  |  |  |
| Sau51 | F: HEX-ACTTCTGATTGCATTGTGAG | AC(10) | | 206-239 | 1 | This study | KX901668 |
|  | R: GTTTGGCAAGATCAGTTTCAC |  | |  |  |  |  |
| Sau53 | F: TET-CATTGGCTCAGAAGTAACAC | AG(8) | | 120-160 | 1 | This study | KX901669 |
|  | R: GTTCTGTATTTGTTTCACAGCC |  | |  |  |  |  |
| Dpu01 | F: TET-TGGATTCACACCCCAAAATT | n/a | | 126-190 | 2 | Dawson et al.*,* 1997 | n/a |
|  | R: AGAAGTATATAGTGCCGCTTGC |  | |  |  |  |  |
| Dpu03 | F: HEX-GAATTACCCATTATTGGATCC | n/a | | 132-236 | 3 | Dawson et al., 1997 | n/a |
|  | R: AGCAGCAAAACAAACCAG |  | |  |  |  |  |
| Dpu16 | F: HEX-ACAGCAAGGTCAGAATTAAA | n/a | | 143-177 | 1 | Dawson et al., 1997 | n/a |
|  | R: AACTGTTGTGTCTGAGCCT |  | |  |  |  |  |
| VeCr02 | F: FAM-AATAGGCTTTGAGGAGGAATCC | (TCA)7 | | 239-275 | 1 | Stenzler et al., 2004 | AY542875 |
|  | R: AGCCCCAAAGTGCTGAAATA |  | |  |  |  |  |
| VeCr06 | F: HEX-TGTCCTCCCCCTGTTTGTTTTA | (ATG)3(TT | | 288-345 | 3 | Stenzler et al., 2004 | AY542879 |
|  | R: ATTGTCCCCACTGCATCCTTCA | G)1(ATG)3 | |  |  |  |  |
| VeCr08 | F: TET-TCACCTCTGATGGGAAATCCTC | (CA)14 | | 161-213 | 3 | Stenzler et al., 2004 | AY542881 |
|  | R: TCACTGGCCTTTGTGTCCATAA |  | |  |  |  |  |
| VeCr10 | F: HEX-CATATACGTGCACCCTCTTCAT | (TTC)6 | | 153-184 | 2 | Stenzler et al., 2004 | AY542882 |
|  | R: TGAGCATTCCTGGTTTCAGATA |  |  |  | |  |  |

**Table S1.2**. Description of microsatellite loci amplified in ovenbird including, sample size (*N*), observed number of alleles (*A*), observed heterozygosity (*H_O_*), expected heterozygosity (*H_E_*), Wright’s inbreeding coefficient (*F_IS_*), populations out of HWE, populations with null alleles and retention in final analyses.

| **Locus** | ***N*** | ***A*** | ***H_O_*** | ***H_E_*** | ***F_IS_*** | **# of populations out of HWE** | **# of populations with nulls** | **Retained in Analysis** |
| --- | --- | --- | --- | --- | --- | --- | --- | --- |
| Sau10 | 336 | 20 | 0.31 | 0.40 | 0.21 | 1 | 2 | Y |
| Sau15 | 321 | 17 | 0.85 | 0.89 | 0.04 | 1 | 1 | Y |
| Sau26 | 314 | 22 | 0.49 | 0.92 | 0.46 | 6 | 5 | N |
| Sau38 | 326 | 8 | 0.34 | 0.65 | 0.48 | 6 | 5 | N |
| Sau45 | 313 | 19 | 0.51 | 0.88 | 0.41 | 5 | 2 | N |
| Sau46 | 300 | 10 | 0.54 | 0.73 | 0.25 | 2 | 4 | N |
| Sau51 | 331 | 21 | 0.82 | 0.83 | 0.00 | 0 | 0 | Y |
| Sau53 | 284 | 14 | 0.24 | 0.73 | 0.66 | 6 | 6 | N |
| Dpu01 | 325 | 31 | 0.82 | 0.95 | 0.12 | 1 | 5 | Y |
| Dpu03 | 327 | 63 | 0.74 | 0.97 | 0.23 | 2 | 5 | N |
| Dpu16 | 338 | 16 | 0.86 | 0.83 | -0.04 | 0 | 0 | Y |
| VeCr02 | 335 | 23 | 0.85 | 0.85 | -0.01 | 0 | 0 | Y |
| VeCr06 | 316 | 18 | 0.83 | 0.88 | 0.05 | 0 | 0 | Y |
| VeCr08 | 323 | 17 | 0.35 | 0.71 | 0.50 | 6 | 6 | N |
| VeCr10 | 338 | 23 | 0.63 | 0.65 | 0.01 | 0 | 0 | Y |

**Table S1.3**. Average pairwise GST matrix from 10 independent subsampled data sets (refer to Fig. S1.4). Values below the diagonal indicate gene flow in a row-to-column direction, whereas values above the diagonal indicate gene flow in a column-to-row direction.

|  | **LSLBO** | **Cypress Hills** | **Black Brook** | **Barachois Pond** | **Gatineau** | **Thunder Bay** |
| --- | --- | --- | --- | --- | --- | --- |
| **LSLBO** | NA | 0.3433 | 0.5512 | 0.5676 | 0.5046 | 0.4677 |
| **Cypress Hills** | 0.4747 | NA | 0.4759 | 0.4812 | 0.4053 | 0.3106 |
| **Black Brook** | 0.5132 | 0.3736 | NA | 0.4958 | 0.6012 | 0.4307 |
| **Barachois Pond** | 0.4792 | 0.2818 | 0.4375 | NA | 0.4597 | 0.5491 |
| **Gatineau** | 0.4901 | 0.4252 | 0.6571 | 0.6498 | NA | 0.4408 |
| **Thunder Bay** | 0.5343 | 0.3136 | 0.5824 | 0.9588 | 0.5074 | NA |

**Figure S1.1**. Log-likelihood plots of L(*K*) showing convergence and optimal choice of *K*. Ten independent runs without sampling information (LOCPRIOR = 0; a) and ten independent runs with sampling information included (LOCPRIOR = 1; b).

**Figure S1.2**. Structure cluster plots. No evidence of genetic differentiation between populations when sampling location was omitted (a) and evidence of weak genetic differentiation of the Cypress Hills population from all other populations when sampling location was included in the analysis (b). Major and minor modes are shown whenever two modes occurred at a specified K.

**Figure S1.3**. Principal coordinates analysis of ovenbird population genetic variation showing three distinct clusters: 1) Cypress Hills; 2) LSLBO, Gatineau, and Black Brook; and 3) Barachois Pond and Thunder Bay. Axis 1 and 2 explain 59.05% and 29.87% of the genetic variation, respectively.

**Figure S1.4.** *G_ST_* networks for the 10 subsampled birds (a-j) from each population showing the strength and direction of gene flow among these populations.
