# Supplementary material for: Phylogeography of a migratory songbird across its Canadian breeding range: Implications for conservation units
Source: Ecol Evol. 2017 Jun 28;7(16):6078–88. doi: 10.1002/ece3.3170 (PMC5574796; doi:10.1002/ece3.3170)
Supplement: Supplementary file 2 [file ECE3-7-6078-s002.docx]

S. Haché, E.M. Bayne, M.-A. Villard, H. Proctor, C.S. Davis, D. Stralberg, J.K. Janes, M.T. Hallworth, K.R. Foster, E. Vasi, A.A. Grossi, J.C. Gorrell, and R. Krikun. Phylogeography of a migratory songbird across its Canadian breeding range: implications for conservation units. *Ecology and Evolution*.

**Appendix S2. Detailed description of the amplification of a fragment of the cytochrome oxidase subunit I (COI) gene, technique used to slide mount 384 syringophilids from 20 host birds, and population structure analyses.**

Following the method described by Dabert et al. (2008), a fragment of the cytochrome oxidase subunit I (COI) gene was amplified by PCR with the following primers: Aseq01F (GGAACRATA TAYTTTATTTTTAGA) and Aseq03R (GGATCTCCWCCTCCWGATGGATT; Glowska et al., 2014). Each 25 uL PCR reaction contained: 5 µl of DNA template, 1X Buffer (10 mM Tris-Cl pH 8.8, 0.1% Triton X-100, 50 mM KCl, 0.16 mg/mL BSA), 0.2 mM dNTP, 1.5 mM MgCl_2_ (Invitrogen), 1.0 µM of each primer and one unit of *Taq* DNA polymerase. Amplification was carried out using a Mastercycler EP thermocycler (Eppendorf). Cycling consisted of an initial denaturation for 5 minutes at 95 °C followed by 35 cycles of 30 seconds at 95 °C, 1 minute at 50 °C, and 1 minute at 72 °C, with a final extension of 5 minutes at 72 °C. PCR products were purified using ExoSAP-IT (Affymetryx) Products were sequenced in both directions using the original amplification primers and BigDye 3.1 chemistry and an ABI3730 sequencer (Applied Biosystems).

Exoskeletons of 384 syringophilids from 20 host birds (see Table S4.1) were recovered from DNA extraction and slide mounted for morphological identification in commercially available poly-vinyl alcohol medium (PVA) (BioQuip Products, Rancho Dominguez, California). Slides were cured for 4 days at ca. 40 °C on a slide-warmer and then examined using Differential Interference Contrast (DIC) optics on a Leica DMLB compound microscope.

**References**

Dabert, J., Ehrnsberger, R. & Dabert, M. (2008) *Glaucalges tytonis* sp. nov. (Analgoidea: Xolalgidae) from the barn owl *Tyto alba* (Strigiformes: Tytonidae): compiling morphology with DNA barcode data for taxa descriptions in mites (Acari). *Zootaxa*, **1719**, 41–52.

[Glowska, E](http://apps.webofknowledge.com/OneClickSearch.do?product=UA&search_mode=OneClickSearch&excludeEventConfig=ExcludeIfFromFullRecPage&SID=3CverHVQhLAhOlyuhxz&field=AU&value=Glowska,%20E)., [Dragun-Damian](http://apps.webofknowledge.com/OneClickSearch.do?product=UA&search_mode=OneClickSearch&excludeEventConfig=ExcludeIfFromFullRecPage&SID=3CverHVQhLAhOlyuhxz&field=AU&value=Dragun-Damian,%20A), A., [Broda](http://apps.webofknowledge.com/OneClickSearch.do?product=UA&search_mode=OneClickSearch&excludeEventConfig=ExcludeIfFromFullRecPage&SID=3CverHVQhLAhOlyuhxz&field=AU&value=Broda,%20L), L., [Dabert](http://apps.webofknowledge.com/OneClickSearch.do?product=UA&search_mode=OneClickSearch&excludeEventConfig=ExcludeIfFromFullRecPage&SID=3CverHVQhLAhOlyuhxz&field=AU&value=Dabert,%20J), J. & [Dabert](http://apps.webofknowledge.com/OneClickSearch.do?product=UA&search_mode=OneClickSearch&excludeEventConfig=ExcludeIfFromFullRecPage&SID=3CverHVQhLAhOlyuhxz&field=AU&value=Dabert,%20M), M. (2014) DNA barcodes reveal female dimorphism in syringophilid mites (Actinotrichida: Prostigmata: Cheyletoidea): *Stibarokris phoeniconaias* and *Ciconichenophilus phoeniconaias* are conspecific. *Folia Parasitologica*, **61**, 272–276.

**Table S2.1**. Mean genetic distance within and among *Betasyringophiloidus* *seiuri* (Clark) based on host location under the K2P model for CO1 (613 bp). Sample size (N) refers to number of host birds from whose quills mite DNA was extracted/number of birds from which mites were slide-mounted for morphological identification.

|  | Cypress Hills | Fort McMurray | LSLBO | Thunder Bay | Gatineau | Black Brook | Barachois Pond | *S. phoeniconaias* |
| --- | --- | --- | --- | --- | --- | --- | --- | --- |
| Cypress Hills (N=2/2) | **0.0270** |  |  |  |  |  |  |  |
| Fort McMurray (N=13/6) | 0.0235 | **0.0190** |  |  |  |  |  |  |
| LSLBO (N= 8/1) | 0.0227 | 0.0178 | **0.0184** |  |  |  |  |  |
| Thunder Bay (N=1/1) | 0.0235 | 0.0146 | 0.0150 | **-** |  |  |  |  |
| Gatineau (N=6/6) | 0.0268 | 0.0180 | 0.0188 | 0.0083 | **0.0146** |  |  |  |
| Black Brook NB (N=7/2) | 0.0221 | 0.0189 | 0.0182 | 0.0157 | 0.0182 | **-** |  |  |
| Barachois Pond (N=1/1) | 0.0329 | 0.0262 | 0.0276 | 0.0191 | 0.0218 | 0.0286 | **0.0185** |  |
| *Stibarokris phoeniconaias* (N=2)^1^ | 0.3773 | 0.3726 | 0.3761 | 0.3789 | 0.3806 | 0.3731 | 0.3708 | **0** |

^1^Number of sequences used from GenBank (KF840699.1 and KF840700.1).


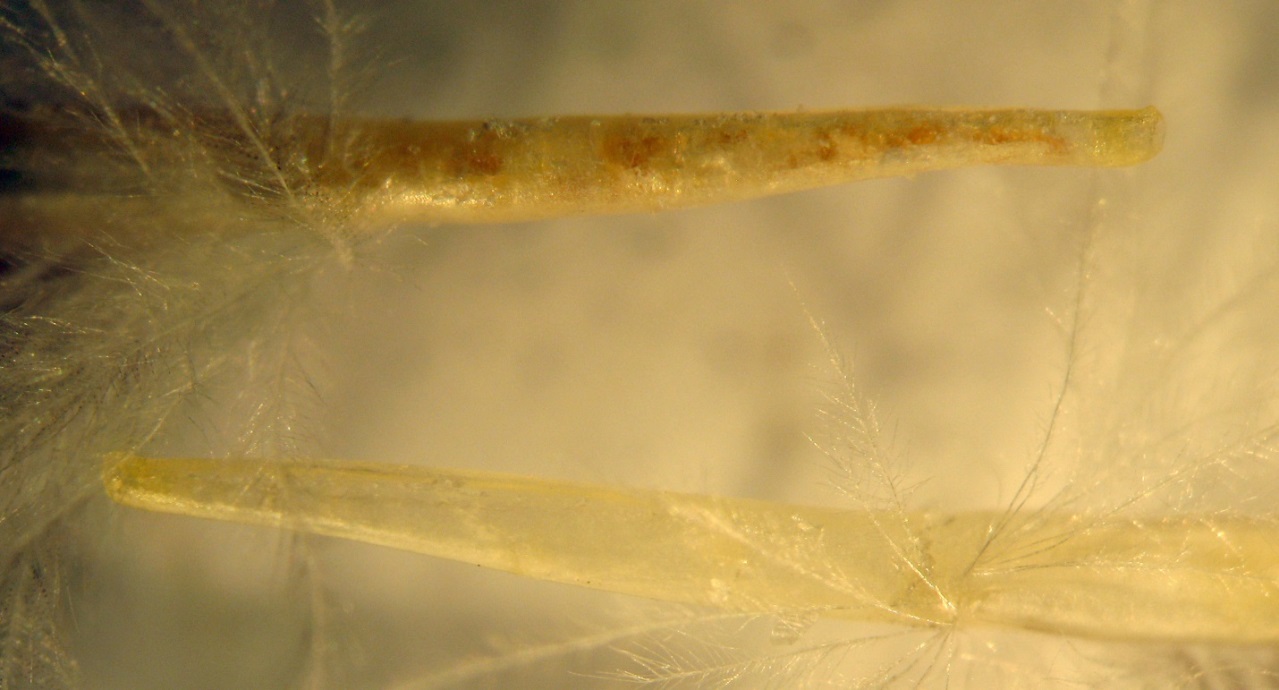


**Figure S2.1**. Quills of Ovenbird tail feathers with (above) and without (below) syringophilid mites.

**
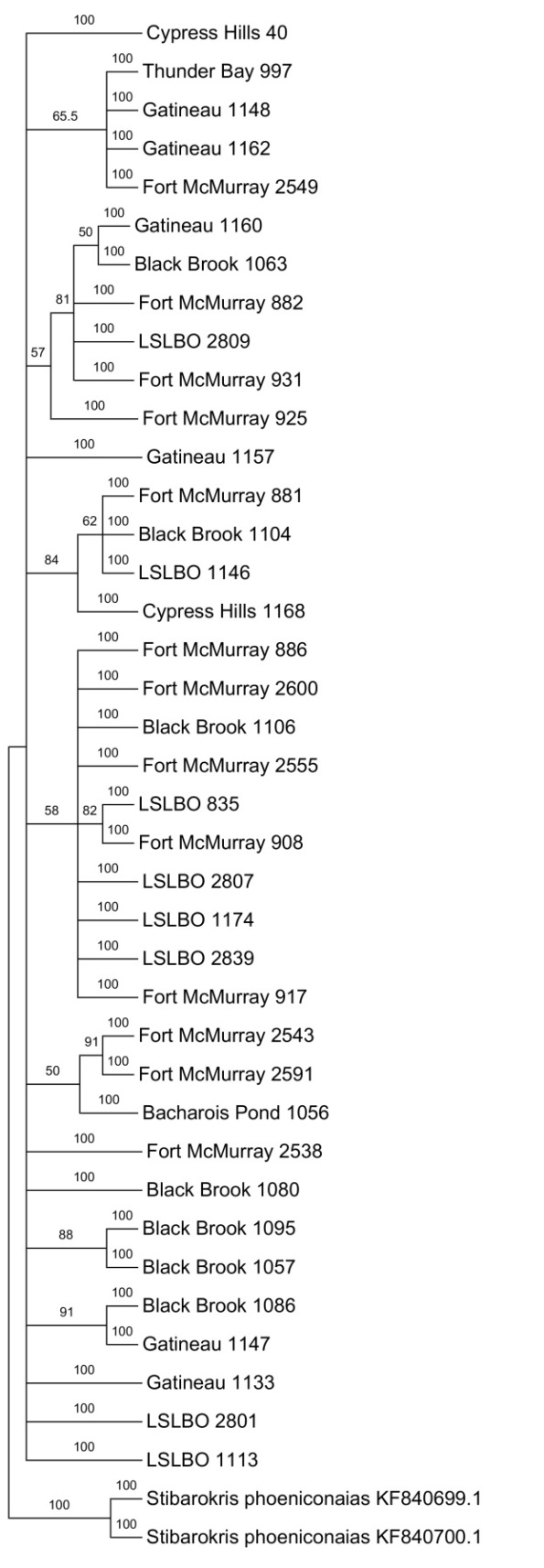
**

**Figure S2.2.** Maximum parsimony analysis of COI (613bp) from *Betasyringophiloidus* *seiuri* (Clark) based on host location. Numbers above branches are bootstrap values (1000 replicates).
