# Supplementary material for: Phylogeography of a migratory songbird across its Canadian breeding range: Implications for conservation units
Source: Ecol Evol. 2017 Jun 28;7(16):6078–88. doi: 10.1002/ece3.3170 (PMC5574796; doi:10.1002/ece3.3170)
Supplement: Supplementary file 3 [file ECE3-7-6078-s003.docx]

S. Haché, E.M. Bayne, M.-A. Villard, H. Proctor, C.S. Davis, D. Stralberg, J.K. Janes, M.T. Hallworth, K.R. Foster, E. Vasi, A.A. Grossi, J.C. Gorrell, and R. Krikun. Phylogeography of a migratory songbird across its Canadian breeding range: implications for conservation units. *Ecology and Evolution*.

**Appendix S3. Light-level geolocator data analysis.**

Light data collected were used to estimate geographic locations (latitude and longitude) using the Solar/Satellite Geolocation for Animal Tracking package (ˋSGATˊ; Wotherspoon et al., 2013; Sumner et al., 2009) in program R. A light threshold of 1 was used to assign the time of sunrise or sunset (transitions). When the light level surpassed this threshold, it was considered a sunrise; the time at which the light level went below was a sunset. We edited or removed sunrise/sunset times that were greater than 45min from sunrise/sunset times within two days on either side of the suspected outlier, and when the sunrise/sunset times of those 4 days occurred within 25min of one another. The SGAT package uses Markov Chain Monte Carlo (MCMC) simulations to estimate geographic locations while incorporating the error inherent in light-level geolocation. We specified a model that included the raw locations derived using the threshold method, a model describing the error distribution between estimated and known sunrise and sunset times, a behavioral model that describes potential flight speeds, and a land mask that constrained stationary periods to land masses. The model was run twice with 2000 iterations on three chains. In between each run, we collapsed the chains and summarized the location estimates. The median daily location was used to initialize the subsequent run. The first two runs were discarded and treated as a burn-in. The final run was composed of 5000 iterations and three chains. We kept every 10^th^ iteration from the posterior distribution from which we drew our geographic inference. Locations drawn from the posterior distribution for which individuals were known to occur on their non-breeding territories (1 November – 1 April) were summarized and was considered to be the non-breeding location. Vernal migration was defined as the interval between departure from the non-breeding grounds and arrival at their breeding locations (after Hallworth et al., 2015).

**References**

Hallworth, M.T., Sillett, T.S., Van Wilgenburg, S.L., Hobson, K.A. & Marra, P.P. (2015) Migratory connectivity of a Neotropical migratory songbird revealed by archival light-level geolocators. *Ecological Applications*, **25**, 336–347.

Sumner, M.D., Wotherspoon, S.J. & Hindell, M.A. (2009) Bayesian estimation of animal movement from archival and satellite Tags. PLoS ONE, **4**, e7324.

Wotherspoon, S.J., Sumner, M.D. & Lisovski, S. (2013) *R Package SGAT: Solar/satellite geolocation for animal tracking*. Available at https://github.com/SWotherspoon/SGAT.
