# Supplementary material for: Phylogeography of a migratory songbird across its Canadian breeding range: Implications for conservation units
Source: Ecol Evol. 2017 Jun 28;7(16):6078–88. doi: 10.1002/ece3.3170 (PMC5574796; doi:10.1002/ece3.3170)
Supplement: Supplementary file 4 [file ECE3-7-6078-s004.docx]

S. Haché, E.M. Bayne, M.-A. Villard, H. Proctor, C.S. Davis, D. Stralberg, J.K. Janes, M.T. Hallworth, K.R. Foster, E. Vasi, A.A. Grossi, J.C. Gorrell, and R. Krikun. Phylogeography of a migratory songbird across its Canadian breeding range: implications for conservation units. *Ecology and Evolution*.

**Appendix S4. Results of paleo-hindcasting by subspecies.**


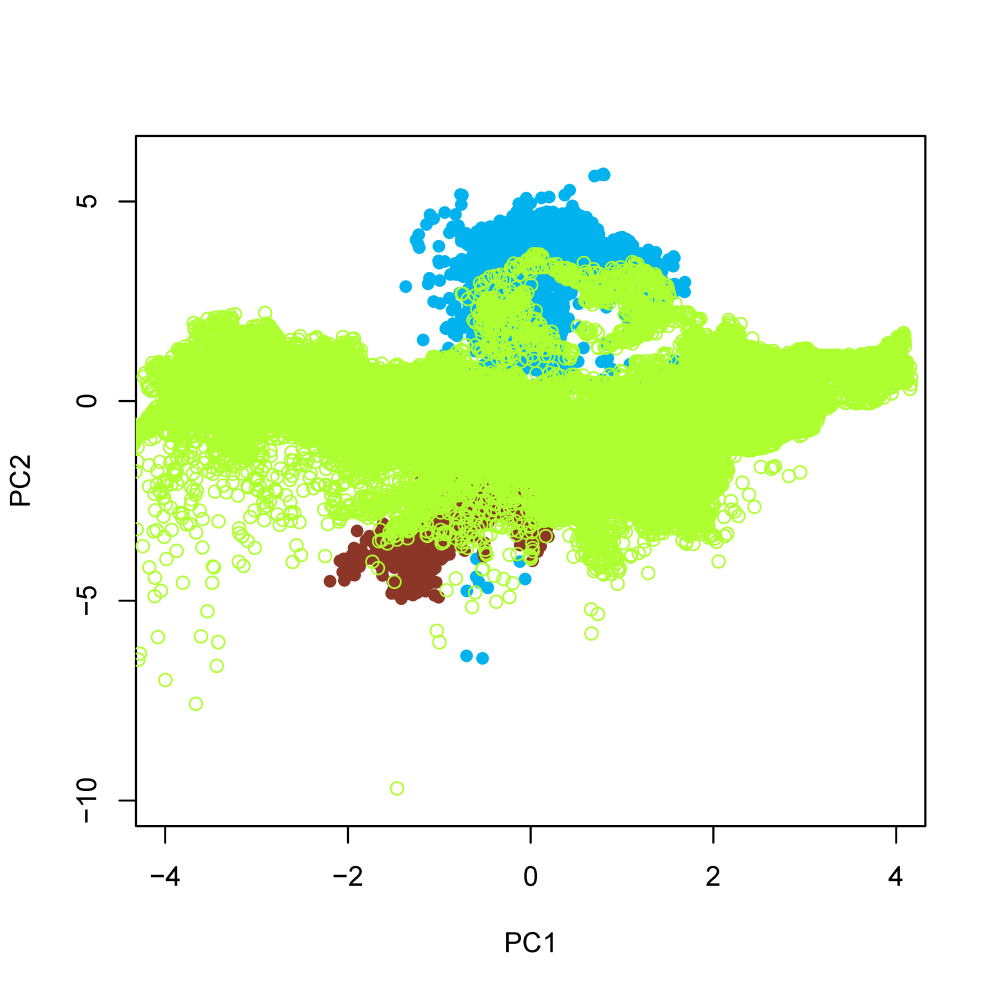


**Figure S4.1**. Plot of climatic niche space of the three mapped Ovenbird (*Seiurus aurocapilla*) subspecies: *S. a. aurocapilla* (green), *S. a. cinereus* (blue) and *S. a. furvior* (maroon). PC1 is the first axis of a principal components analysis (PCA) explaining 56% of the variance and representing cold winter temperatures. PC2, the second axis of a PCA, explains an additional 34% of overall variance, and represents dry summer conditions.


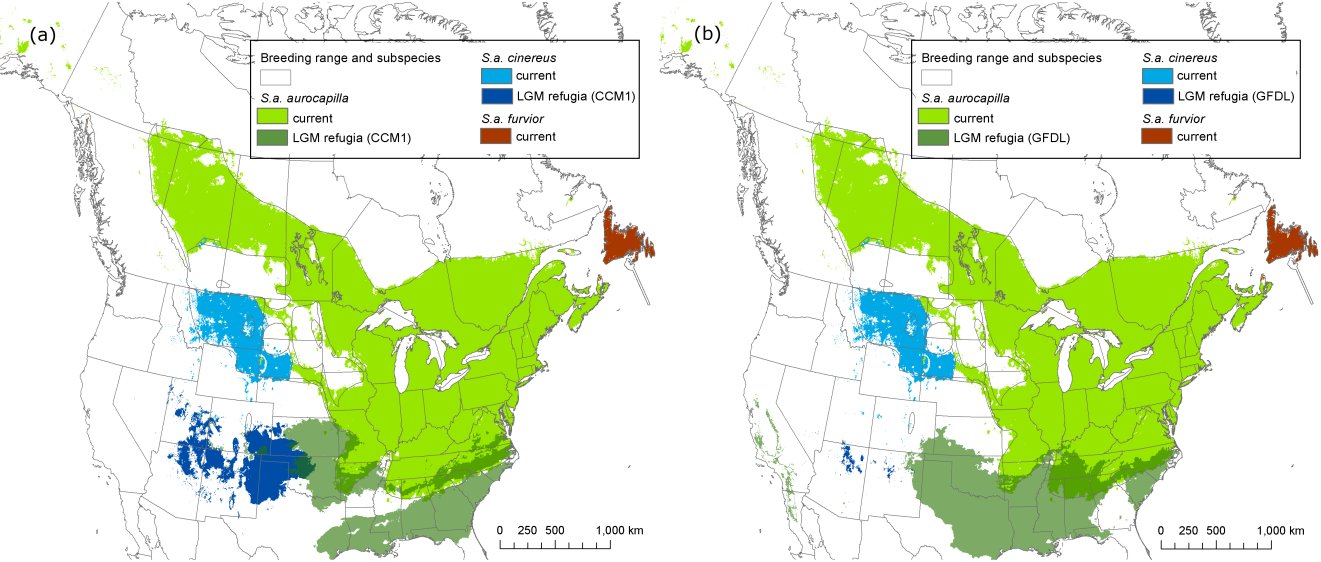


**Figure S4.2.** Projected climatic suitability of breeding ranges for each Ovenbird subspecies during last glacial maximum based on two U.S. global climate models (Community Climate Model [CCM1; A] and Geophysical Fluid Dynamics Laboratory [GFDL; B] model). Climatic suitability was determined using a threshold of 0.5 probability of occurrence.
